# Subfossil trees suggest enhanced Mediterranean hydroclimate variability at the onset of the Younger Dryas

**DOI:** 10.1038/s41598-018-32251-2

**Published:** 2018-09-18

**Authors:** Maren Pauly, Gerhard Helle, Cécile Miramont, Ulf Büntgen, Kerstin Treydte, Frederick Reinig, Frédéric Guibal, Olivier Sivan, Ingo Heinrich, Frank Riedel, Bernd Kromer, Daniel Balanzategui, Lukas Wacker, Adam Sookdeo, Achim Brauer

**Affiliations:** 1 GFZ German Research Centre for Geosciences, Section 5.2 Climate Dynamics and Landscape Evolution, Potsdam, Germany; 2 Free University Berlin, Department of Earth Sciences, Section of Palaeontology, Berlin, Germany; 3 Aix Marseille Univ, Avignon Université, CNRS, IRD, IMBE, Mediterranean Institute of Marine and Terrestrial Biodiversity and Ecology, Aix-en-Provence, France; 4 University of Cambridge, Department of Geography, Cambridge, United Kingdom; 5 Swiss Federal Institute for Forest, Snow and Landscape Research WSL, Dendrosciences, Birmensdorf, Switzerland; 6 Global Change Research Centre and Masaryk University, Brno, Czech Republic; 7 French National Institute for Preventive Archaeological Research, Venelles, France; 8 University of Heidelberg, Institute of Environmental Physics, Heidelberg, Germany; 9 ETH Zürich, Ion Beam Physics, Zürich, Switzerland; 10 University of Potsdam, Institute for Earth and Environmental Science, Potsdam, Germany; 11 Humboldt-University Berlin, Geography Department, Berlin, Germany

## Abstract

Nearly 13,000 years ago, the warming trend into the Holocene was sharply interrupted by a reversal to near glacial conditions. Climatic causes and ecological consequences of the Younger Dryas (YD) have been extensively studied, however proxy archives from the Mediterranean basin capturing this period are scarce and do not provide annual resolution. Here, we report a hydroclimatic reconstruction from stable isotopes (δ^18^O, δ^13^C) in subfossil pines from southern France. Growing before and during the transition period into the YD (12 900–12 600 cal BP), the trees provide an annually resolved, continuous sequence of atmospheric change. Isotopic signature of tree sourcewater (δ^18^O_sw_) and estimates of relative air humidity were reconstructed as a proxy for variations in air mass origin and precipitation regime. We find a distinct increase in inter-annual variability of sourcewater isotopes (δ^18^O_sw_), with three major downturn phases of increasing magnitude beginning at 12 740 cal BP. The observed variation most likely results from an amplified intensity of North Atlantic (low δ^18^O_sw_) versus Mediterranean (high δ^18^O_sw_) precipitation. This marked pattern of climate variability is not seen in records from higher latitudes and is likely a consequence of atmospheric circulation oscillations at the margin of the southward moving polar front.

## Introduction

During the abrupt and intense climate change from the Allerød warm phase to the YD cold reversal in the North Hemisphere (ca. 12 700–11 600 cal BP)^1,2^ sea-ice production and drifting enhanced^3^, alpine glaciers advanced^4^, storm intensity strengthened^5^, and a reorganization of the atmosphere^6,7^ may have occurred. Greenland ice core data (NGRIP) reveal temperature drops of 10–15 °C with simultaneous reductions in snow accumulation and amplifications in atmospheric dust within less than a decade^6,8^. During the rapid cooling, lake sediment records across Europe signal intensified wind stress, aridity and detrital input, alongside drastic ecological changes^5,9,10^. The results are spatially heterogeneous in terms of hydrological change, as other European lake records find more humid conditions and/or increased quantity and intensity of precipitation (associated with higher lake levels in certain cases)^11,12^. Model simulations constrained by proxy data indicate no single factor could cause the observed YD cold reversal, but rather a complex combination of weakened Atlantic meridional overturning circulation (AMOC), altered atmospheric circulation patterns, and moderate negative radiative forcing as most plausible driving factors^13^. Nevertheless, mechanisms of climate variability in the North Atlantic region remain under intense debate^8^, even for the most recent past^14^.

In the Mediterranean, climate information on the YD from terrestrial records is scarce. Distinct change is evident in speleothem δ^18^O (Chauvet cave^15^), although a significantly less pronounced drop in summer temperatures (July) than at mid-latitudes has been reported^16^. Today, the Mediterranean climate is characterized by hot-dry summers, and relatively mild (depending on orography)-humid winters (Fig. S1). Model simulations specify that this summer-dry/winter-wet regime also persisted at the Last Glacial Maximum (LGM) when climate-forcing mechanisms were substantially different^17^. Thus, we assume that this general feature of seasonality was active during the Allerød/YD transition and in particular cold season precipitation, snow storage, and subsequent spring melt provided the water source for the studied trees.

**Figure 1:**
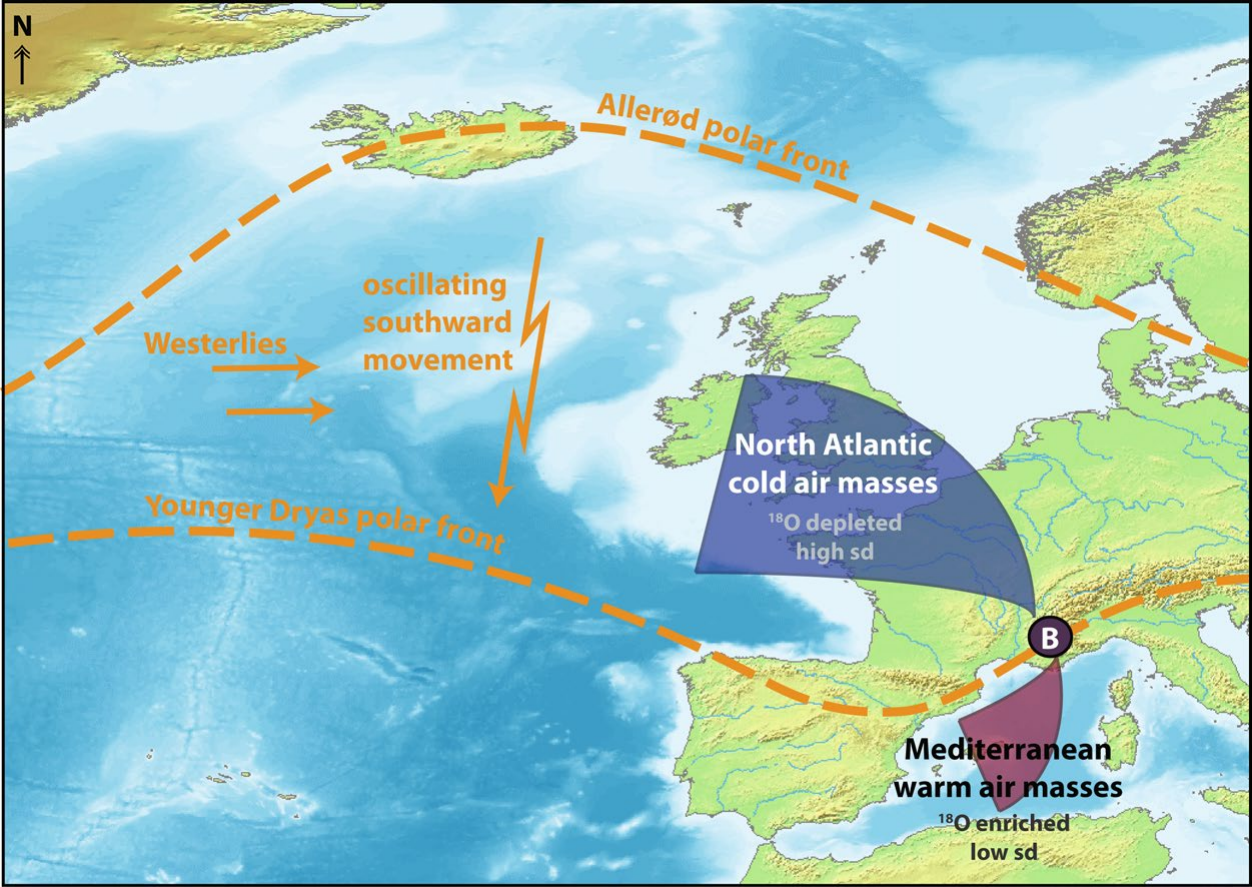
Influences of air mass conversions and the oscillating polar front on Barbiers during the Late Glacial: Map indicating position of Barbiers (B) in conjunction with hypothesized polar front variability (from Allerød to Younger Dryas)^36,37^ and influential air masses (North Atlantic vs. Mediterranean)^19^. Map produced in Illustrator, using a Wikimedia Commons public domain base map from DEMIS Mapserver (https://commons.wikimedia.org/wiki/File:WorldMap-B_non-Frame.png).

The study site is located in the western Mediterranean on the foothills of the southern French Alps (Barbiers region: 44°21′11″N, 5°49′50″E, Fig. S2)^18^. The steeply sloped valley of Barbiers shows evidence of highly unstable geomorphological conditions, where the subfossil trees were discovered enclosed (and thus well-preserved) within an alluvial sediment deposit, caused by multiple flooding events^18^. The region surrounding Barbiers is situated within a transitional climatic zone that is influenced by warm Mediterranean, cool Atlantic and mixing of air masses from both origins. Generally, precipitation from North Atlantic air masses is characterized by rather low, but highly variable δ^18^O values, whereas vapour produced over the Mediterranean sea carries a higher δ^18^O signature with low variance^19^. During the YD, when the Polar Front migrated south (Fig. 1), the interaction and mixing of these air masses was conceivably more intense and frequent in southern France. Although evident at the global scale^20^, Mediterranean oxygen isotopes of precipitation (δ^18^O_precip_) do not show strong relationships to local surface temperatures, as they are more strongly influenced by origin of moist air masses, transport lengths, rainout histories and amount of precipitation^19^. Hence, δ^18^O_precip_ at Barbiers predominantly signals changes in the relative contribution of precipitation from air masses of the two different origins (Fig. 1), an assumption that provides the theoretical basis for the Polar Front interpretation in this study.

Here we present carbon and oxygen isotope chronologies from tree-ring cellulose (δ^13^C_cell_, δ^18^O_cell_) used to develop the first annually resolved, biochemical climate proxy for reconstructing the abrupt cooling transition to the YD in the Mediterranean, thereby extending the latitudinal transect of annually-resolved records southward from Greenland^6^ and western Germany^5^. The records were built using a subset of seven well preserved trees from a floating tree ring-width chronology^18,21^, of relatively low replication (9 trees) as sub-fossil trees growing during the Allerød/YD transition remain elusive^22^. The individual trees of the chronology have relatively short average lifespans between 95–210 years, indicative of the highly variable and unstable site conditions. Despite the short-lived trees, none of the known ontogenetic effects related to age or tree height^23,24^ were evident in the stable isotope data (Fig. S5). Tree-ring width patterns are in some cases concurrent with tree burying in this region (abrupt growth decrease)^18^ and thus may not record a clear climate signal, particularly in consideration of the low sample replication.

**Figure 2:**
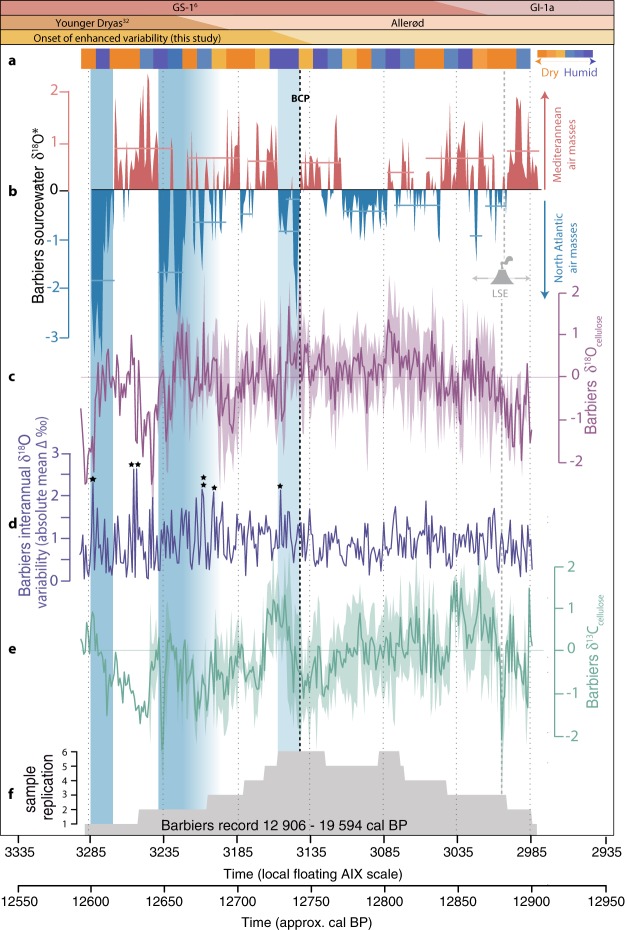
Barbiers tree-ring stable isotopes and palaeoclimate proxy records: (**a**) dual-isotope model recording dry vs. humid phases (10-year steps), derived from stable carbon and oxygen isotope ratios^21^; (**b**) modelled Barbiers sourcewater δ^18^O (‰ vs. VSMOW, z-scored, see Methods); mean z-scored tree cellulose (**c**) δ^18^O (‰ vs. VSMOW), (**d**) inter-annual variability thereof (mean absolute change, ‰ – asterisks designate extremes of >2‰), (**e**) δ^13^C (‰ vs. VPDB) and (**f**) sample replication. Laacher See Eruption (LSE)^5,30^ indicated with 40-year dating error. Blue shaded areas highlight periods of extreme sourcewater depletion during the period of enhanced inter-annual variability (Barbiers Change Point, BCP).

The entire underlying cross-dated tree-ring series (9 trees) was positioned on the absolute time scale by ^14^C wiggle matching with Kauri tree-ring data proposed by Capano *et al*.^21,25^ (Methods, Fig. S2). Our reconstructions (7 trees) date 12 906–12 594 cal BP and cover 312 years (Fig. 2). In a multi-parameter approach, tree ring-width, δ^18^O_cell_ and δ^13^C_cell_ (Fig. S3) were utilized in combination with NGRIP δ^18^O-derived annually resolved temperature^6^ to (a) reconstruct local sourcewater δ^18^O (δ^18^O*_SW_, predominately reflecting oxygen isotopes of precipitation; Fig. 2b and (b) to estimate relative humidity, both based on leaf-level dual-isotope theory^26,27^ (Fig. 2a and S4, Methods). Annual NGRIP δ^18^O was positioned by synchronization to annual (varved) Meerfelder Maar lake sediment records (via the Vedde Ash Tephra), hence the Barbiers tree-ring chronology was positioned on the absolute time scale independently (Methods, Fig. S2)^21^.

**Figure 3:**
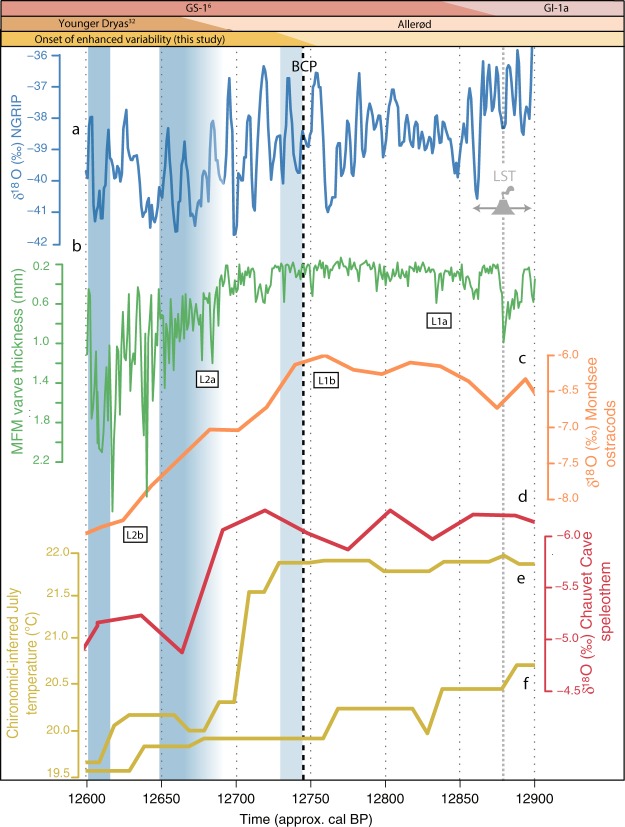
Selected palaeoclimate proxy records in the Northern Hemisphere: (**a**) NGRIP δ^18^O (‰ vs. VSMOW)^6^; Meerfelder Maar (**b**) varve thickness^33^; (**c**) Mondsee ostracod δ^18^O (‰ vs. VPDB)^29^; (**d**) Chauvet Cave speleothem δ^18^O (‰ vs. VPDB)^15^; chironomid inferred July air temperatures^16^ in (**e**) the Alpine region and (**f**) southwest Europe. Position of Laacher See Tephra (LST, 12 880 ± 40 varve years BP)^30^ and event timing of GS-1 onset at NGRIP (deuterium excess), Meerfelder Maar (L1a: aquatic lipid biomarkers, L2a: varve thickness), Mondsee (L1b: ostracod δ^18^O, L2b: increased NAP). Blue shaded areas indicate periods of extremely depleted modelled sourcewater δ^18^O values at Barbiers (this study). See Figure S6 for map of selected records.

During the first few decades of the Barbiers record (12 906 to 12 865 cal BP), the trees show increases in δ^18^O_cell,_ with stable or increasing δ^13^C_cell_ and isotopically-heavy (modelled) sourcewater (Fig. 2bce)–indicative of the Late Glacial climate improvement following the Gerzensee Oscillation (Switzerland)^9^, GI-1b (Greenland)^28^ or Mo-LG3 (Austria)^29^. Embedded in this transition period from humid/cooler to drier/warmer conditions is a sharp, short-lived (5-year) decline in δ^13^C_cell_ (n_trees_ = 2), concurrent yet less pronounced increase in δ^18^O_cell_ (n_trees_ = 1) corresponding with the timing of the Laacher See volcanic eruption (LSE) within dating uncertainties (Fig. 2: LSE, Fig. 3: LST, 12 880 ± 40 cal BP, Meerfelder Maar^5,30^). This volcanic eruption likely produced a stratospheric volcanic plume capable of reaching southern France^31^, resulting in reduced solar radiation (from increased atmospheric opacity), with the potential to induce a short-term reduction in photosynthetic efficiency and/or stomatal conductance, as reflected in the δ^13^C_cell_ record.

The tree-ring isotope records continue relatively constantly for the next 160 years (12 865 to 12 740 cal BP), until an increase in inter-annual variability of δ^18^O_cell_ (+0.2‰ absolute, Fig. 2d) and δ^18^O*_SW_ (0.87‰ to 2.13‰, Fig. 2b, Table S2) coincides with an enhanced magnitude and frequency of extreme years (doubling of events with >3.0‰ inter-annual difference), particularly in pulses of isotopically light sourcewater (values exceeding 2 standard deviations beyond the mean, Fig. 2b, Table S1) occurring from from 12 740 cal BP; hereafter referred to as the Barbiers Change Point (BCP). The influence of sample replication on changes in variability of the mean time series was tested using two segments with equally low sample replication (2 trees) pre- (12 906–12 883 cal BP) and post- (12 680–12 642 cal BP) BCP. This analysis yielded average absolute inter-annual variabilities of 0.61‰ and 1.24‰, respectively; proving low sample replication is likely not a cause of the increase, yet is still a limitation in the dataset. Despite the initial significant, short-lived sourcewater depletion at the BCP boundary, change point analysis (Methods) suggests five distinct δ^18^O*_SW_ change phases; the first in variability (12 702 cal BP) and the following four in mean (>0.5‰: 12 664, 12 646, 12 616, 12 608 cal BP) (Table S2). This provides further evidence that the transformation to extreme conditions (BCP) occurred within an overall switch to new conditions according to the mean chronology.

We argue that the conversion of air masses formed at mid- and high- latitudes (Fig. 1) with those from the Mediterranean intensified in southern France at the onset of the YD (BCP), along the margin of the southward moving polar front; producing more intense cold season storms from both origins (enhanced δ^18^O*_SW_ variability) and more frequent and/or more intense precipitation events (progressively increasing magnitude of negative δ^18^O*_SW_ excursions) originating from North Atlantic air masses. The positive δ^18^O*_SW_ excursions after the BCP (particularly between 12640–12615 cal BP, Table S2) may be related to more frequent Mediterranean tropical-like cyclones (MTLCs), that are fostered by strong vertical temperature gradients between the sea surface and high troposphere. Together with a general southward shift of cold season lows over mid latitudes, more frequent upper atmospheric cold intrusions meeting warm and moist low pressure systems over the western Mediterranean sea is likely, increasing the number of MTLCs and worsening of the growing conditions (storm and flood damage) for the trees at Barbiers. However, these positive excursions are, however, lower amplitude than the negative excursions associated with increased polar outbreak and cold extremes (Tables S1, S2).

At 12 593 cal BP a final local tree die-off occurs, conceivably reflecting the reduction in *Pinus* forests and expansion of shrub vegetation found across Europe^9,29^. This corroborates with evidence of reduced competition and forest thinning from the overall negative lifespan trend in δ^13^C_cell_ (n_trees_ = 5, Fig. S5) and an increase in photosynthetic efficiency (δ^13^C_cell_) of the last remaining tree.

In central Europe, two-step sequential YD transitions have been identified in annually laminated records from Meerfelder Maar^30,32^ (Fig. 3: L1a, L2a) and Mondsee (Fig. 3: L1b, L2b; Fig. S6)^29^. The initial transition step (decline of lipid biomarker δD (L1a) and calcite δ^18^O (L1b), respectively) has been attributed to the onset of decreasing temperatures. Whereas the second step was interpreted as a consequence of enhanced storminess and aridity, as seen in sediment regime expressed as varve micro-faces change^5^ and vegetation alterations and a lake level drop: increased varve thickness at Meerfelder Maar (L2a) and reduced calcite precipitation/increased flux of allochthonous sediments at Mondsee (L2b). This is contradictory to results at Barbiers, where the climate change was not one-directional (i.e. continuous alteration in atmospheric regime to glacial conditions), but rather bi-directional with enhanced extremes in both humidity and precipitation. Along these lines, the continuous speleothem growth at Chauvet Cave (130 km from Barbiers, Fig. S6) across the onset of the YD is in contrast with speleothems located at higher latitudes (i.e. Villars Cave), which show reduced growth or hiatus^15^ signalling persistent low relative humidity during the YD cold reversal (as found at Meerfelder Maar^31^). From BCP onwards, drops in Barbiers δ^18^O*_SW_ are simultaneous with deuterium excess in the NGRIP record^6^, a proxy for North Atlantic moisture-source evaporative conditions. In contrast, the δ^18^O*_SW_ upswing phases at Barbiers are not recorded in the North Atlantic, Meerfelder Maar or Mondsee, hinting that they are an expression of a locally specific climate anomaly (i.e. phases of intensified precipitation (e.g. MTLCs) originating from the Mediterranean Sea). Together, evidence of a latitudinal discrepancy in the Mediterranean becomes clear, where increased magnitude/frequency of precipitation events (this study) and relatively high humidity^15^ were prevalent rather than enhanced aridity as often recorded north of the Alps^29,30^.

The temperature decline elucidating the cold reversal (L1a, L2a, GS-1) is still evident (yet more gradual) in lower latitude (Mediterranean-influenced) speleothem^15^ and sediment core^11,33^ records, and thus likely at Barbiers (though this is intertwined within the δ^18^O_SW_ signal). When considering the mean Barbiers tree δ^18^O_cell_ record alone, a general decline of 2‰ is evident from 12 740 cal BP onward. Since this represents a complex signal of paired sourcewater and physiological dynamics, it is only through the proxy climate reconstruction (sourcewater and relative humidity) that this signal can be interpreted in detail as a complex signal of air mass origin, transportation, conversion and resultant storm tracks; rather than simply deduced as a stable drop in temperature. The contrasting sourcewater signature of strengthened MTLCs (enriched δ^18^O*_SW_) versus the higher frequency of polar outbreaks (depleted δ^18^O*_SW_) in the Mediterranean may explain the delayed and/or lower amplitude YD cooling traced in available sediment records within the region^11,15,33^; the coarse resolution of which would dampen the signal of enhanced inter-annual variability in both directions. Further, the highly resolved Meerfelder Maar data recorded a brief, decadal oscillation in varve facies and pollen preceding this distinct YD transition^32^, underscoring the importance of high resolution records in reconstructing incremental/progressive change during climate instability and change, as seen in the step-wise oscillatory nature of hydroclimate variability in southern France at Barbiers.

The isotope data presented here suggest the importance of the careful consideration of spatial disparities when comparing multiple records of past rapid climate oscillations across a vast region, as slight latitudinal differences can coerce divergent feedback mechanisms associated with complex atmospheric and oceanic circulation changes. This study provides new insight into the behaviour of sub-fossil trees from annually-resolved stable isotope data during the intense climate change of the Late Glacial, and proves the potential of combined tree-ring parameters (ring-width, stable carbon and oxygen isotopes) in reconstructing local hydrological dynamics resulting from changing atmospheric circulation. We find further evidence to support the theorized southern movement of the polar front^5,34^, expressed as an enhanced amplitude and frequency of winter storms and extreme events at Barbiers during the onset of a widespread and probably more capricious than previously thought reversal to the near glacial conditions of the YD.

## Methods

### Sampling, chronology development and radiocarbon dating

Subfossil pine trunks (*Pinus sylvestris* L., n_trees_ = 18: 16 *in-situ*, 2 uprooted) were collected adjacent to the Barbiers riverbed in southeastern France (44°21′11″N, 5°49′50″E, Figs S2, S7) from an alluvial deposit spread across three tributaries on the southern foothills of Saint-Genis Mountain^18,35^. Cross-sectional disks were cut and their surfaces were polished using various sandpapers (80 to 1200 grit sizes) to assist with tree-ring identification. Tree-ring width was measured on numerous tracks per disk using a LINTAB measuring table combined with TSAP-Win software. Samples were initially radiocarbon dated at low-resolution^18,35^, within 1σ ^14^C error range, and tree-ring width series of all trees were cross-dated visually and statistically (TSAP-Win) to build two initial floating chronologies (BarbA n_trees_ = 6, BarbB n_trees_ = 3, Fig. S3a,b)^18^; 9 additional trees discovered (younger and older than BarbA and BarbB) could not be cross-dated and thus were not included in the chronologies. Subsequently, the chronologies were radiocarbon dated with high-resolution ^14^C analyses performed at CEREGE (Centre de Recherche et d’Enseignement de Géosciences de l’Environnement) Aix-Marseille University^21^. The new high-resolution radiocarbon (^14^C) dates of a two-tree sequence (210 years: Barb-12, Barb-17, Fig. S3) were measured using every 3^rd^ ring within each tree^21^; previous ^14^C dating (to build BarbA and BarbB) was based on 10-year blocks^18,35^. The new sequence (used to connect BarbA and BarbB into a single chronology) was wiggle matched with the decadal Kauri^25^ and YDB^36^ chronologies using visual tuning. This analysis permitted the inclusion of Barb-17 and Barb-5 (both initially BarbB) into the BarbA chronology, where initial tree placement results^18^ required secondary confirmation, as there was more than one statistically plausible cross-dated position. A sequence age of 12 836 to 12 594 cal BP was found for the Barb12-Barb17 sequence, thus allowing the connection of the two tree-ring width chronologies into one^21^ and placing the entire floating dendroisotope chronology (this study) at 12 906 to 12 594 cal BP (Fig. S3).

Chronological synchronization between the three annually resolved data sets from Greenland (NGRIP^6^), western Germany (Meerfelder Maar^37^) and southern France (Barbiers, this study) was completed using (1) volcanic tie points (Vedde Ash^38^) and (2) radiocarbon wiggle matching^21^. Meerfelder Maar is renowned for its high chronological precision, with continuous annually laminated sediments from the Late Glacial to the Holocene and three tephra layers (isochrons) in the Holocene (Ulmener Maar Tephra, 11 000 varve BP^37,39^), Younger Dryas (Vedde Ash, 12 140 varve BP^38^) and Allerod (Laacher See Tephra, 12 880 varve BP^37^). Direct evidence from the Vedde volcanic eruption (Vedde Ash = VA) has also been conclusively discovered in NGRIP^40^, providing a tie point to synchronize the two annual records. Based on these assumptions, the NGRIP VA (12 171 ± 144 b2k) has been matched to Meerfelder Maar VA (12 140 ± 40 varve BP)^41^, with a shift of 19 years (12 190 b2k) applied to the data utilized in this study.

Since the dendroisotope record at Barbiers is older than the Vedde eruption and shows no direct evidence of the Laacher See eruption (i.e. local volcanic ash *in-situ*), it was independently placed on the calendar scale based on results by Capano *et al*.^21^. The known dates of Meerfelder Maar tephra isochrones and Barbiers trees permit the estimation of absolute ages, allowing a robust connection between the three proxy archives and thus a regional inter-site comparison of climatic events.

### Stable isotope analysis

A subset of samples (n = 7, 12 906–12 594 cal BP, Fig. S3) with high preservation and clear tree ring boundaries was selected for stable isotope analysis. One track on each tree disk was cut from pith to bark with a conventional band saw and then radially sliced into 1–1.5 mm width ‘thick’ sections (modified Isomet 5000 precision saw, Buehler, Esslingen, Germany). Individual annual rings were separated by hand using a scalpel blade for cellulose extraction. Holocellulose was extracted from wholewood using the two-step base-acid method^42,43^: sodium hydroxide for resin and extractives removal followed by acidified sodium chlorite to eliminate lignins. Following extraction, samples were washed thoroughly with milli-Q water, homogenized (ultrasonic sonode device for Eppendorf sample vials) and then freeze-dried for 48 hours. Resultant homogenized cellulose was weighed and packed in silver (tin) capsules for stable oxygen (carbon) analysis. Measurements were completed on an Isotope Ratio Mass Spectrometer Delta V, ThermoFisher Scientific, Bremen, Germany with TC/EA HT pyrolysis device for δ^18^O determination (Isotope Ratio Mass Spectrometer ISOPRIME coupled online to a Carlo Erba NA1500 Elemental Analyzer for δ^13^C). The samples analyzed are referenced to standard materials from the International Atomic Energy Agency (IAEA-C3, IAEA-CH6, IAEA-601 and IAEA-602), and checked with secondary standards from Sigma-Aldrich Chemie GmbH, Munich, Germany (Sigma Alpha-Cellulose and Sigma Sucrose) using a two-point normalization method^44^. Sample replication resulted in a reproducibility of better than± 0.1‰ for δ^13^C_cell_ values and± 0.3‰ for δ^18^O_cell_ values. The isotope ratios are given in the δ-notation, relative to the standards V-PDB for δ^13^C and V-SMOW for δ^18^O (Fig. S4).

### Proxy reconstruction calculations of local sourcewater (δ^18^O_SW_) and relative humidity

Local sourcewater δ^18^O signature (δ^18^*O_SW_, Fig. 1b) was calculated based on the model of Anderson *et al*.^45^:

1 $${\delta }_{{\rm{s}}{\rm{w}}}={\delta }^{18}{{\rm{O}}}_{{\rm{c}}{\rm{e}}{\rm{l}}{\rm{l}}}{\textstyle \text{-}}\,(1-{\rm{f}})(1-{\rm{r}}{\rm{H}})({\varepsilon }_{{\rm{e}}}+{\varepsilon }_{{\rm{k}}})-{\varepsilon }_{{\rm{b}}{\rm{i}}{\rm{o}}{\rm{c}}{\rm{h}}{\rm{e}}{\rm{m}}}$$

The dampening factor (f) was calculated as per Equation 2^45^ and relative humidity from Equation 3^46^:

2 $${\rm{f}}=-\,1.47{\rm{rH}}+0.03{\rm{T}}+0.11{\rm{TRX}}+0.62$$

3 $${{\rm{\delta }}}^{13}{{\rm{C}}}_{{\rm{cell}}}=(\,-\,0.17){\rm{rH}}+(-0.15){\rm{T}}-6.0$$

Temperature (T) was derived from annual NGRIP ice core δ^18^O^6^ (calibration of T = δ^18^O + 3‰^47^), with latitudinal (+50 °C) and growing season (+8 °C) corrections and the Tree Ring Index (TRX) was calculated for individual trees and then averaged into the mean chronology. Constants of ε_biochem_ = 27‰^48–50^, ε_e_ = 28‰^51^ and ε_k_ = 28‰^52^ were used. The statistical influence of NGRIP δ^18^O (as a predictor for temperature) on the modelled sourcewater has been approximated to test the impact of chronological error. Regression models were used to calculate the relative importance of multiple input variables (δ^18^O_NGRIP_, δ^18^O_cell_, δ^13^C_cell_, tree ring width: Figs 1, S4) on the modelled sourcewater output. The calculated linear model coefficients of the dendrodata (δ^18^O_cell_, δ^13^C_cell_, tree-ring width) were two orders of magnitude higher (0.674, −0.539 and 0.393, respectively) than NGRIP data (−0.006), proving the dual-isotope model output is stable within dating uncertainties. These results are logical as δ^18^O_cel_ is mainly a measure of local sourcewater variability, influenced by stomata conductance (also recorded in δ^13^C_cell_), which is driven by relative humidity and thus temperature (inherent in tree ring widths).

The resultant δ^18^*O_SW_ was subtracted from δ^18^O_cell_ to extract the proportion of δ^18^O_cell_ changes due to changes in (leaf level) vapour pressure (∆δ^18^O_cell-SW_) over sourcewater; utilized in a dual-isotope modelling approach^21^ to infer periods of high and low humidity, by comparing decadal-scale trajectories (+/−) of ∆δ^18^O_cell-SW_ and δ^13^C (Fig. S4).

Inter-annual δ^18^O_cell_ variability (Fig. 2d, ∆‰) was calculated by subtracting current year δ^18^O_cell_ (‰) from previous year δ^18^O_cell_ (‰) to calculate ‰ difference per year of each individual tree. These values were then converted to absolute differences and subsequently averaged to produce a mean curve (∆‰).

Change point analysis was completed using an R package (‘changepoint’)^53^ to find the position of multiple change points within the modelled sourcewater time series (δ^18^*O_SW_) according to mean and variability; with four and one change points found, respectively (Table S2).

## Electronic supplementary material

Supplementary Information

## Data Availability

The datasets generated during the current study are available from the corresponding author upon reasonable request.
