# Supplementary material for: Subfossil trees suggest enhanced Mediterranean hydroclimate variability at the onset of the Younger Dryas
Source: Sci Rep. 2018 Sep 18;8:13980. doi: 10.1038/s41598-018-32251-2 (PMC6143623; doi:10.1038/s41598-018-32251-2)
Supplement: Supplementary file 1 — Supplementary Information [file 41598_2018_32251_MOESM1_ESM.pdf]

Supplementary Information

Subfossil trees likely reveal enhanced Mediterranean hydroclimate variability at the onset of the Younger Dryas

Maren Pauly[1,2]*, Gerhard Helle[1,2], Cécile Miramont[3], Ulf Büntgen[4,5,6], Kerstin Treydte[5], Frederick Reinig[5], Frédéric Guibal[3], Olivier Sivan[7], Ingo Heinrich[1], Frank Riedel[2], Bernd Kromer[8], Daniel Balanzategui[1], Lukas Wacker[9], Adam Sookdeo[9], Achim Brauer[1,10]

mpauly@gfz-potsdam.de

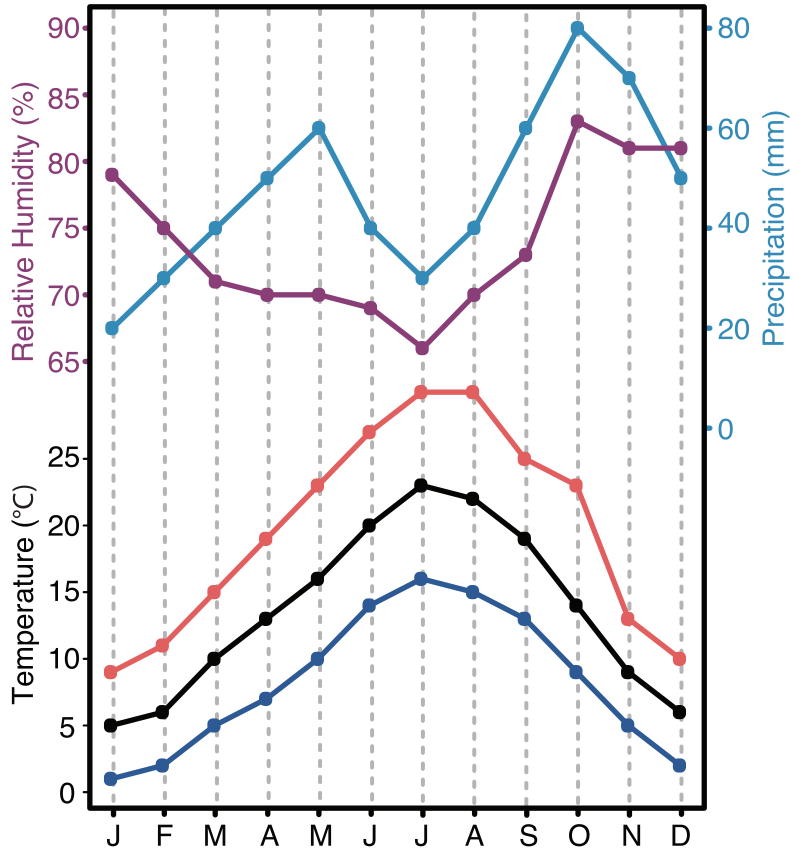

S1 Present day climate indices: annual variability in relative humidity, precipitation, and temperature (red = high, black = average, blue = low) at nearby climate station (Avignon, France).

a	BARB-4	BARB-5	BARB-7	BARB-12	BARB-13	BARB-14	BARB-17
BARB-4	- / 0	2.9 / 95	2.1 / 107	5.7 / 139	4.5 / 87	4.5 / 156	1.4 / 55
BARB-5	2.9 / 95	- / 0	4.0 / 92	3.2 / 95	1.1 / 17	0.1 / 86	2.0 / 40
BARB-7	2.1 / 107	4.0 / 92	- / 0	3.9 / 131	0.5 / 14	0.5 / 83	0.7 / 79
BARB-12	5.7 / 139	3.2 / 95	3.9 / 131	- / 0	2.4 / 46	3.0 / 115	3.8 / 126
BARB-13	4.5 / 87	1.1 / 17	0.5 / 14	2.4 / 46	- / 0	8.4 / 104	- / 0
BARB-14	4.5 / 156	0.1 / 86	0.5 / 83	3.0 / 115	8.4 / 104	- / 0	0.1 / 31
BARB-17	1.4 / 55	2.0 / 40	0.7 / 79	3.8 / 126	- / 0	0.1 / 31	- / 0

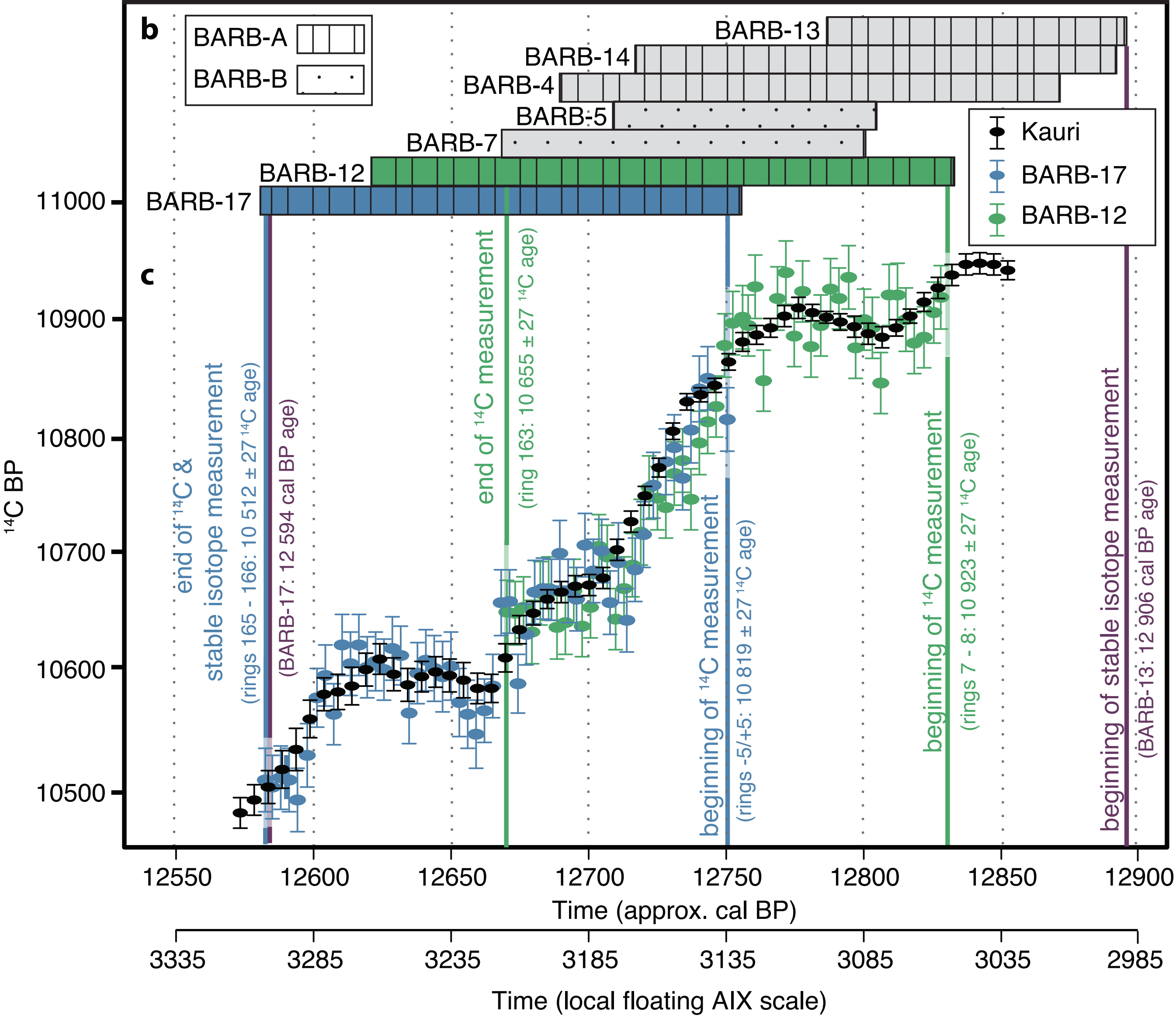

Figure S2. Cross-dating and radiocarbon wiggle matching of Barbiers trees: (a) Statistical cross-dating of seven Barbiers trees utilized for the dendroisotope record (this study), based on results from refs 22 and 25. (T-Value with Baillie-Pilcher-Standardization / number of overlapping years); (b) overlap of individual trees from two tree chronologies (BarbA, BarbB)[22]; (c) trees wiggle matched with Southern Hemisphere (Kauri) decadal radiocarbon calibration curve[24] using high resolution ^{14}C measurements from a two-tree sequence (Barb 12 & Barb 17, as per ref 25); Kauri ^{14}C ages corrected for the inter-hemispheric gradient (57 ^{14}C years subtracted)

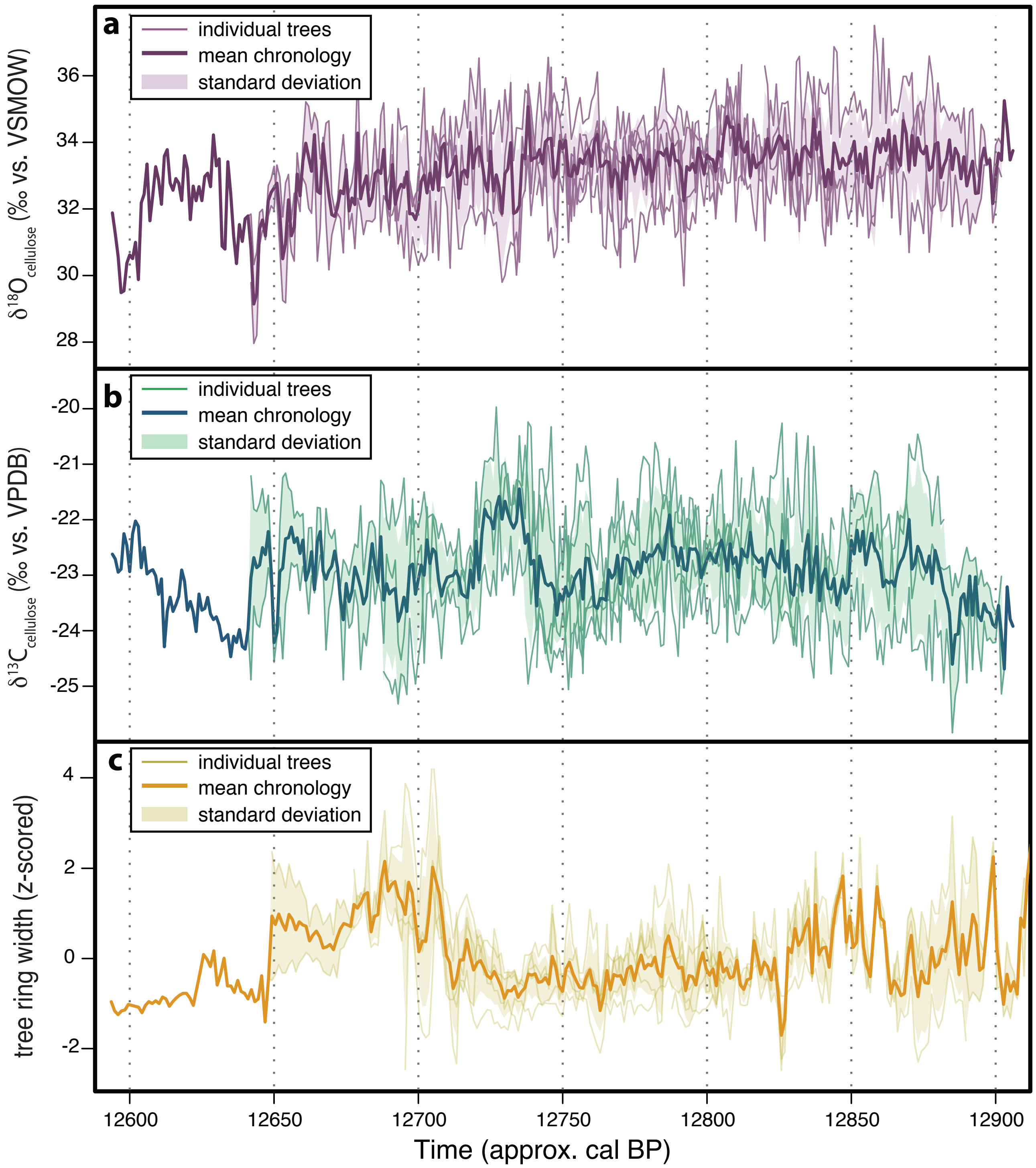

Figure S3. Tree-ring parameters of Barbiers trees: (a) oxygen ($\delta^{18}O_{cel}$ ‰ vs. VSMOW) and (b) carbon ($\delta^{13}C_{cel}$ ‰ vs. VPDB), measured at the GFZ Helmholtz Centre Potsdam, Section 5.2 Climate Dynamics and Landscape Evolution and (c) tree-ring widths (z-scored), measured at CEREGE, Aix-Marseille University.

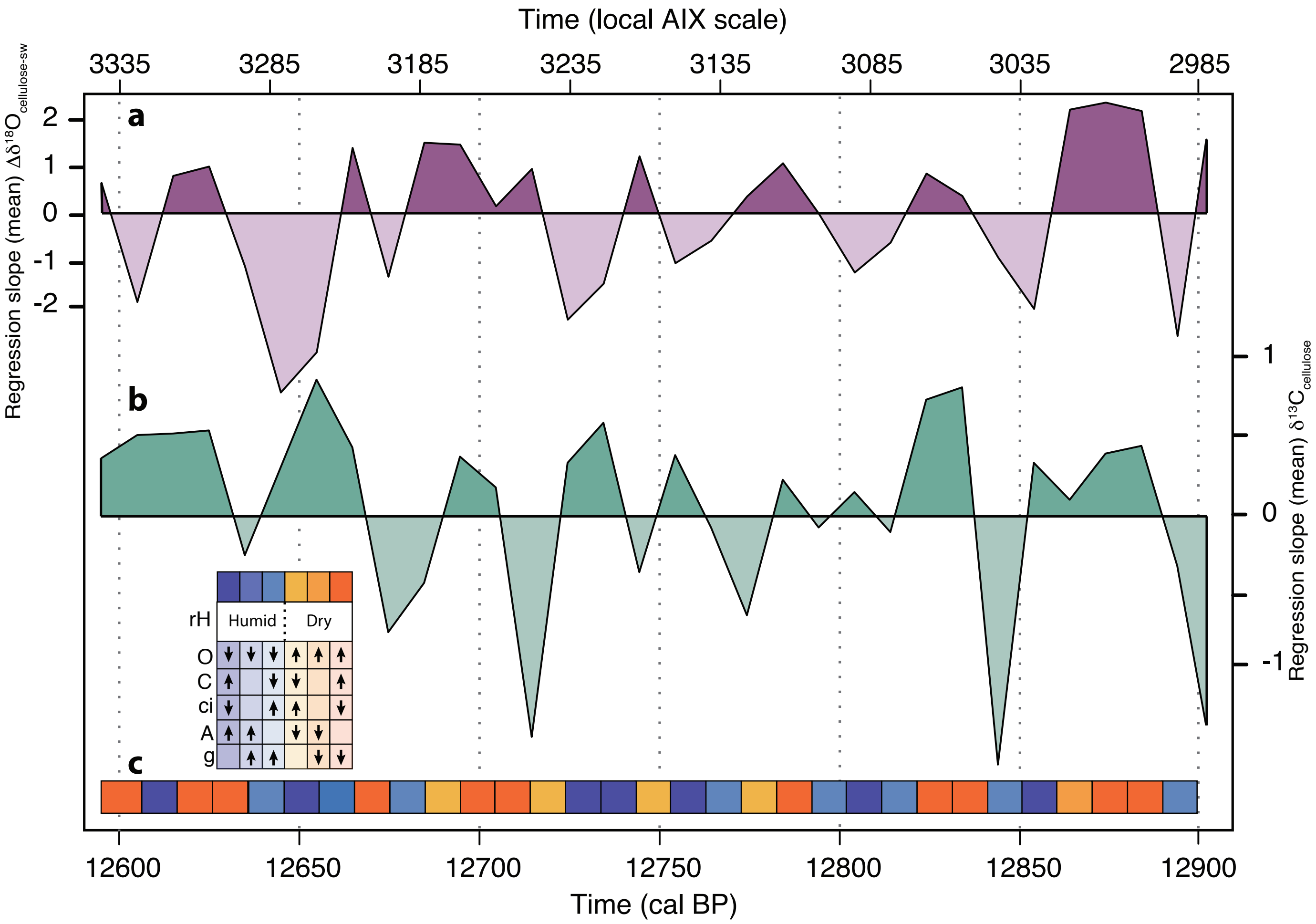

Figure S4. Dual-isotope modelling results of Barbiers trees: Decadal regression slopes from mean (a) $\delta^{18}O$ (stomata-driven $\delta^{18}O$ signal: $\Delta\delta^{18}O = \delta^{18}O$cellulose - $\delta^{18}O$sw) and (b) $\Delta\delta^{13}C$ records. Resultant $\Delta\delta^{18}O$-$\delta^{13}C$ scenarios[16] plotted (c). Table inset: relative humidity (rH) and CO_2 leaf partial pressure (Ci) derived from $\delta^{18}O$ (O) and $\delta^{13}C$ (C) trajectory (a,b), respectively. Maximum photosynthesis (A) and stomatal conductance (g) change output based on aforementioned parameters.

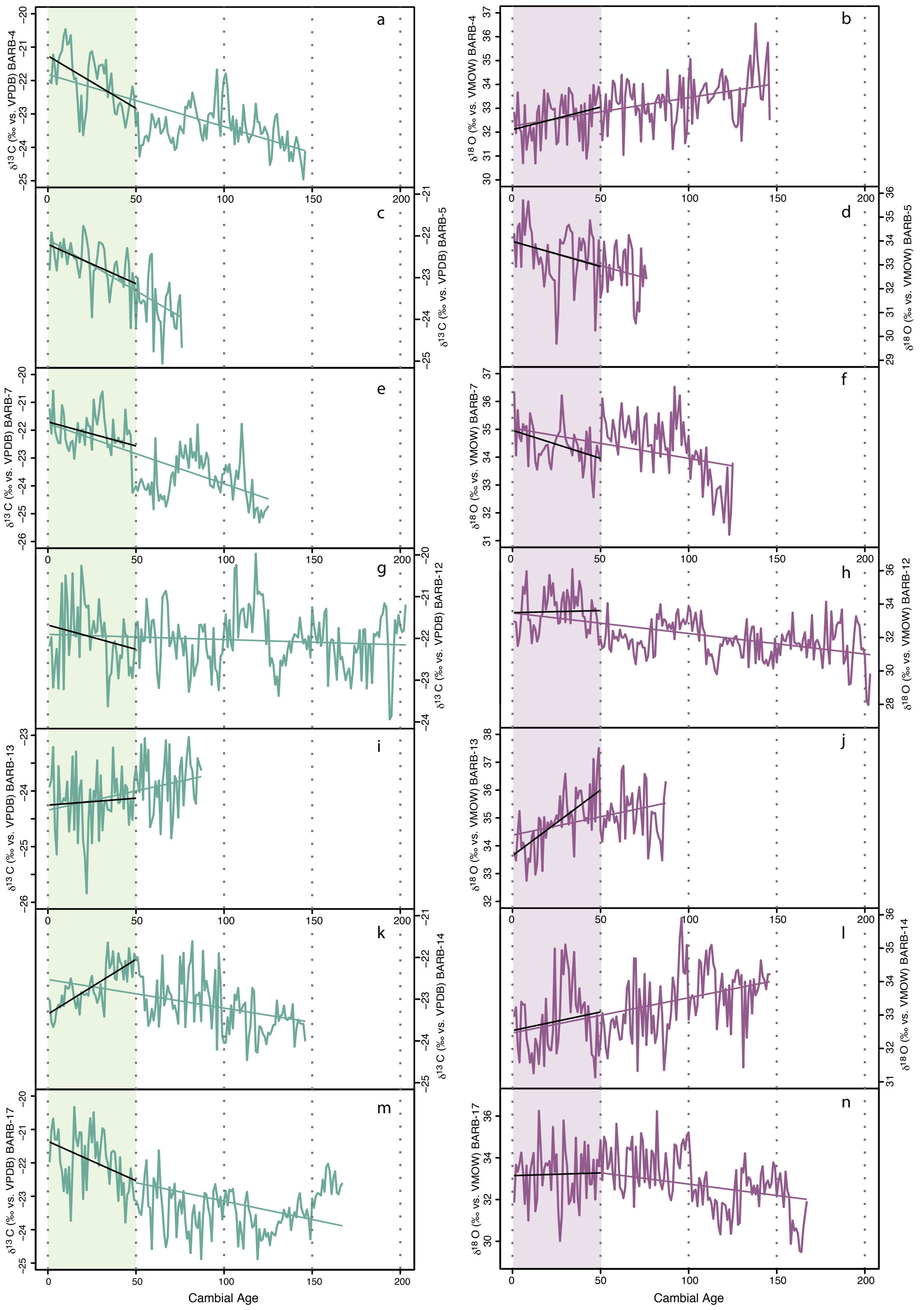

Figure S5. Individual dendroisotope records of Barbiers River trees: Age trends in carbon (green, ‰ vs. VPDB) and oxygen (purple, ‰ vs. VSMOW) isotopes from (a,b) BARB-4, (c,d) BARB-5, (e,f) BARB-7, (g,h) BARB-12, (i,j) BARB-13, (j,l) BARB-14, (m,n) BARB-17 for first 50 years (juvenile years, black lines) and full tree (green/purple lines). Innermost rings (at most 10) may be missing due to preservation issues.

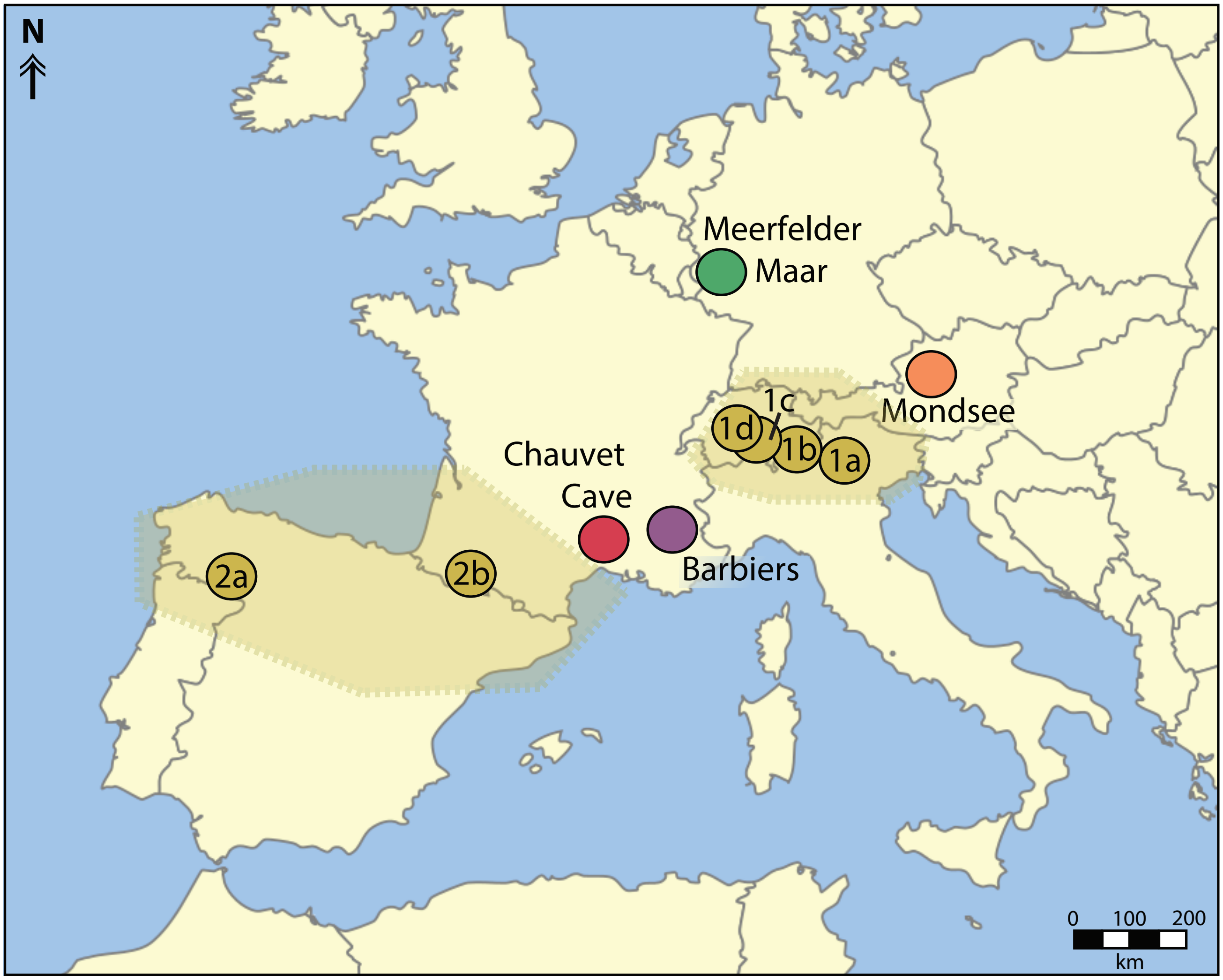

Figure S6. Location of selected palaeoclimate proxy records within Europe: Barbiers subfossil trees (this study), Chauvet Cave speleothems[15], Mondsee[29] and Meerfelder Maar[30,32] indicated. Locations of chironomid-inferred July temperatures[16] in the Alpine Region (1a: Lago di Lavarone, 1b: Maloja Riegel, 1c: Foppe, 1d: Hinterburgsee) and southwest Europe (2a: Laguna de la Roya, 2b: Ech). Highlighted areas for each temperature proxy region shows estimated spatial area represented by the stacked record.

Time range	Average ‰ sourcewater	Number of years
12 740 – 12 738 cal BP	-3.3‰	3
12 662 – 12 658 cal BP	-3.6‰	5
12 653 – 12647 cal BP	-3.4‰	7
12 640 – 12639 + 12 636 cal BP	+2.6‰	3
12 608 – 12 601 cal BP	-4.6‰	8

Table S1: Periods of enhanced sourcewater depletion (-) and enrichment (+), with values greater than 2 standard deviations beyond the chronology mean.

Change Points	
mean	12 664 cal BP
	12 646 cal BP
	12 616 cal BP
	12 608 cal BP
variability	12 702 cal BP

Table S2: Change points defined in the sourcewater time series according to mean and variability using 'changepoint' R package (Killick R and Eckley IA, 2014).
